# Cardiomyocyte-Specific Expression of Lamin A Improves Cardiac Function in *Lmna* ^−/−^ Mice

**DOI:** 10.1371/journal.pone.0042918

**Published:** 2012-08-15

**Authors:** Richard L. Frock, Steven C. Chen, Dao-Fu Da, Ellie Frett, Carmen Lau, Christina Brown, Diana N. Pak, Yuexia Wang, Antoine Muchir, Howard J. Worman, Luis F. Santana, Warren C. Ladiges, Peter S. Rabinovitch, Brian K. Kennedy

**Affiliations:** 1 Department of Biochemistry, University of Washington, Seattle, Washington, United States of America; 2 Department of Pathology, University of Washington, Seattle, Washington, United States of America; 3 Department of Physiology and Biophysics, University of Washington, Seattle, Washington, United States of America; 4 Department of Comparative Medicine, University of Washington, Seattle, Washington, United States of America; 5 Department of Pharmacology and Physiology, University of Rochester, Rochester, New York, United States of America; 6 Buck Institute for Age Research, Novato, California, United States of America; 7 Department of Medicine and Department of Pathology and Cell Biology, College of Physicians and Surgeons, Columbia University, New York, New York, United States of America; Brigham and Women's Hospital, United States of America

## Abstract

*Lmna* ^−/−^ mice display multiple tissue defects and die by 6–8 weeks of age reportedly from dilated cardiomyopathy with associated conduction defects. We sought to determine whether restoration of lamin A in cardiomyocytes improves cardiac function and extends the survival of *Lmna* ^−/−^ mice. We observed increased total desmin protein levels and disorganization of the cytoplasmic desmin network in ∼20% of *Lmna* ^−/−^ ventricular myocytes, rescued in a cell-autonomous manner in *Lmna* ^−/−^ mice expressing a cardiac-specific lamin *A* transgene (*Lmna* ^−/−^; Tg). *Lmna* ^−/−^; Tg mice displayed significantly increased contractility and preservation of myocardial performance compared to *Lmna* ^−/−^ mice. *Lmna* ^−/−^; Tg mice attenuated ERK1/2 phosphorylation relative to *Lmna* ^−/−^ mice, potentially underlying the improved localization of connexin43 to the intercalated disc. Electrocardiographic recordings from *Lmna* ^−/−^ mice revealed arrhythmic events and increased frequency of PR interval prolongation, which is partially rescued in *Lmna* ^−/−^; Tg mice. These findings support our observation that *Lmna* ^−/−^; Tg mice have a 12% median extension in lifespan compared to *Lmna* ^−/−^ mice. While significant, *Lmna* ^−/−^; Tg mice only have modest improvement in cardiac function and survival likely stemming from the observation that only 40% of *Lmna* ^−/−^; Tg cardiomyocytes have detectable lamin A expression. Cardiomyocyte-specific restoration of lamin A in *Lmna* ^−/−^ mice improves heart-specific pathology and extends lifespan, demonstrating that the cardiac pathology of *Lmna* ^−/−^ mice limits survival. The expression of lamin A is sufficient to rescue certain cellular defects associated with loss of A-type lamins in cardiomyocytes in a cell-autonomous fashion.

## Introduction

Nuclear lamins are type V intermediate filament proteins that are implicated in a variety of cellular processes, including DNA replication, gene transcription and chromatin organization [1], [2], [3], [4]. Mutations within the A-type lamin gene, *LMNA*, are associated with over 13 different tissue-specific diseases, collectively termed laminopathies. These include autosomal Emery-Dreifuss muscular dystrophy (EDMD2/3), limb-girdle muscular dystrophy type 1B (LGMD1B), and dilated cardiomyopathy with conduction defects 1A (CMD1A). Adipose, bone, and neural tissues can also be affected in laminopathies, which may resemble aspects of accelerated or premature aging (for review see Worman, et al. 2010) [5], [6].

Mice lacking the *Lmna* gene appear normal at birth but progressively display multiple tissue defects, including muscular dystrophy and dilated cardiomyopathy, with a noticeable reduction in growth rate beginning as early as 2 weeks of age followed by premature death at 6–8 weeks [7]. Further analysis of hearts from *Lmna* ^−/−^ mice has revealed a rapid development of left ventricular dilatation coupled with decreased systolic function beginning after 2 weeks of age [8]. Dosage of A-type lamins can also influence cardiac function as *Lmna^+/−^* mice display cardiac conduction defects with a late onset of dilated cardiomyopathy [9]. However, expression of either major isoform alone, lamin A only [10] or lamin C only [11], is sufficient to prevent phenotypes observed in *Lmna* ^−/−^ mice, indicating that either isoform can largely compensate for the other. Interestingly, only homozygous—but not heterozygous—knock-in mouse models for either muscular dystrophy-associated or cardiac-specific *LMNA* mutations [12], [13] display dilated cardiomyopathy with conduction defects and premature death. In contrast, humans heterozygous for the corresponding missense mutations [14], [15] develop cardiac and skeletal muscle pathology, indicating that there are subtle differences in disease manifestation between rodents and humans.

In this study, we tested whether cardiomyocyte-specific expression of lamin A [16] can improve cardiac function and increase lifespan of *Lmna* ^−/−^ mice. We show better preservation of myocardial performance and reduced occurrence of conduction abnormalities for *Lmna* ^−/−^ mice expressing the cardiac transgene. These observations are consistent with a partial restoration of localization and protein levels of desmin, connexin43 and ERK1/2 phosphorylation. The heterogenic expression of the cardiac lamin A transgene in *Lmna* ^−/−^ hearts underlies this partial restoration and limits lifespan extension, however, with this model we are able to investigate cell-autonomous and non-cell autonomous roles which lends insight into the biology of A-type lamins in the cardiac system.

## Results

### Cardiac-specific expression of FLAG-tagged human lamin A in *Lmna* ^−/−^ mice

**Figure 1:**
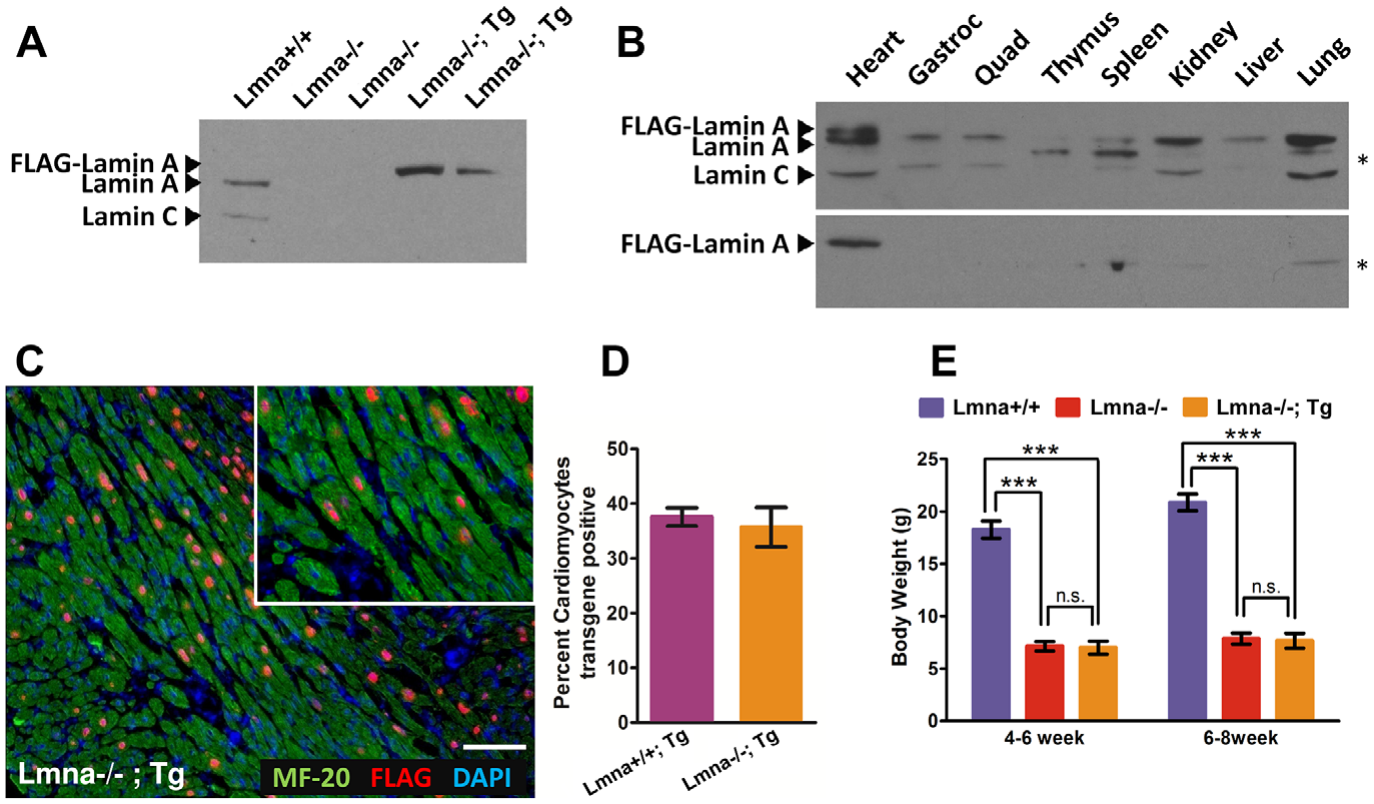
FLAG-lamin A is highly expressed, tissue-specific, and mosaic. (A) Western blot analysis of *Lmna* ^+/+^, *Lmna* ^−/−^, and *Lmna* ^−/−^; Tg heart lysates that were probed with lamin A/C antibody. (B) Western blot analysis of heart, gastrocnemius, quadriceps, thymus, spleen, kidney, liver, and lung from *Lmna* ^+/+^; Tg (upper panel) and *Lmna* ^−/−^; Tg (lower panel) mice probing with lamin A/C antibody. The asterisk indicates a cross-reactive band present in all lanes. (C) Indirect immunofluorescence micrograph of *Lmna* ^−/−^; Tg heart cross-section stained for MF-20 (cardiomyocytes; green), FLAG (transgene; red), and DAPI (nuclei, blue). Note that the mosaic expression of the transgene is limited to only cardiomyocytes. Scale bar denotes 100 µm. Inset is zoomed in view from the same image. (D) Quantitation of the percent of cardiomyocytes expressing FLAG-lamin A (N = 3 each; ∼500 cardiomyocytes were scored for each group) (E) Body weights of *Lmna* ^+/+^, *Lmna* ^−/−^, and *Lmna* ^−/−^; Tg mice (N = 10, 11, and 8 at 4–6 weeks respectively and N = 10, 7, and 5 at 6–8 weeks respectively).

To determine whether cardiac-specific expression of lamin A can improve heart function in *Lmna* ^−/−^ mice, we crossed transgenic mice expressing FLAG-tagged human lamin A under the α-myosin heavy chain promoter [16] with *Lmna* ^+/−^ mice to ultimately produce litters containing both *Lmna* ^−/−^ mice and *Lmna* ^−/−^ mice expressing the cardiac-specific *LMNA* transgene (*Lmna* ^−/−^; Tg). FLAG-lamin A is highly expressed in *Lmna* ^−/−^; Tg cardiac tissue as measured by Western analysis using an A-type lamin antibody **(** **Figure 1A** **),** and expression is specific to cardiac tissue in both *Lmna* ^+/+^; Tg and *Lmna* ^−/−^; Tg mice **(** **Figure 1B** **)**. Indirect immunofluorescence microscopy using antibodies against myosin heavy chain (MF-20) and FLAG **(** **Figure 1C** **)** indicate that ∼35% of ventricular myocytes express the FLAG-lamin A transgene in both *Lmna* ^+/+^; Tg and *Lmna* ^−/−^; Tg hearts **(** **Figure 1D** **)**. Finally, expression of the cardiac-specific transgene in *Lmna* ^−/−^; Tg mice does not improve the growth defect of *Lmna* ^−/−^ mice as measured by their body weights compared to *Lmna* ^+/+^ mice **(** **Figure 1E** **)**. Collectively, these results show that the FLAG-lamin A transgene is highly – though mosaically – expressed and specific to cardiomyocytes.

### Characterization of molecular phenotypes associated with cardiac structure and remodeling in *Lmna* ^−/−^ and *Lmna* ^−/−^; Tg hearts

**Figure 2:**
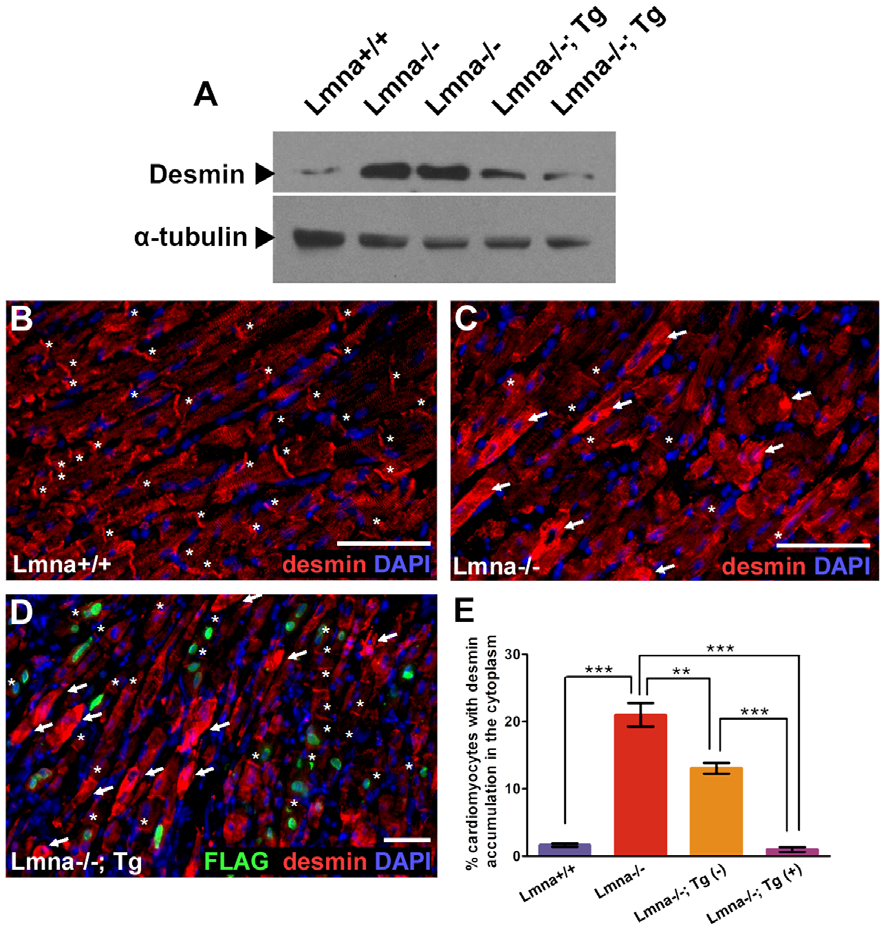
Transgenic expression of lamin A in *Lmna* ^−/−^ cardiomyocytes results in partial restoration of desmin phenotype but not cardiac remodeling markers. (A) Western blot analysis of *Lmna* ^+/+^, *Lmna* ^−/−^, and *Lmna* ^−/−^; Tg whole heart lysates. Desmin is increased in *Lmna* ^−/−^ heart lysates compared to *Lmna* ^+/+^ and is partially attenuated in *Lmna* ^−/−^; Tg mice (middle blot). α-tubulin is shown as a loading control (bottom blot). (B–D) Indirect immunofluorescence micrographs showing desmin (red), FLAG (green), and DAPI (blue) in *Lmna* ^+/+^ (B), *Lmna* ^−/−^ (C), and *Lmna* ^−/−^; Tg (D) heart ventricle sections. Asterisks mark intercalated discs, which co-localize with a pan-cadherin antibody (not shown) and arrows indicate ventricular myocytes with accumulated desmin in the cytoplasm. Scale bar denotes 50 µm. (D) Note the lack of ventricular myocytes positive for both desmin cytoplasmic accumulation and transgene expression. (E) Graph summarizing the desmin cytoplasmic accumulation phenotype observed in *Lmna* ^−/−^ ventricular myocytes across different mouse backgrounds aged 5–7 weeks (N = 3 for *Lmna* ^+/+^, *Lmna* ^−/−^, and *Lmna* ^−/−^; Tg; ** P<0.01; *** P<0.001).

Western analysis was performed on lysates derived from hearts of 5–7 week old *Lmna* ^+/+^ *Lmna* ^−/−^, and *Lmna* ^−/−^; Tg mice to determine whether molecular phenotypes are restored in the presence of cardiac lamin A expression. Desmin, previously determined to be mislocalized in *Lmna* ^−/−^ cardiomyocytes [8], exhibits an approximately 3-fold increase in total protein levels from *Lmna* ^−/−^ hearts **(** **Figure 2A** **)**. This finding in cardiomyocytes contrasts with skeletal muscle myoblasts from *Lmna* ^−/−^ mice, where desmin levels are reduced [17]. Expression of FLAG-lamin A in the heart results in attenuation of total desmin that is only approximately 2-fold increased compared to *Lmna* ^+/+^ hearts. Desmin is mislocalized in *Lmna* ^−/−^ ventricular myocytes, resulting in diminished staining in the majority of intercalated discs **(** **Figures 2B** **vs. 2C)** as well as increased cytoplasmic desmin staining in ∼21% of ventricular myocytes (P<0.001) **(** **Figure 2E** **)**. Less than 1% of transgene-expressing ventricular myocytes from *Lmna* ^−/−^; Tg mice display increased cytoplasmic desmin (P<0.001) **(** **Figures 2D and 2E** **)**, suggesting cell-autonomous rescue of desmin localization. Due to the mosaic nature of our transgene expression, we continued to observe increased cytoplasmic desmin in the transgene-non-expressing ventricular myocytes, but also noted a 38% reduction in the fraction of these cells, possibly due to neighboring transgene-expressing ventricular myocytes (P<0.01; see Discussion). These findings are consistent with the increase of desmin in hearts of *Lmna* ^−/−^ mice and the partial attenuation of desmin levels in *Lmna* ^−/−^; Tg mice.

### Improved cardiac contractile function in transgenic *Lmna* ^−/−^ mice

**Figure 3:**
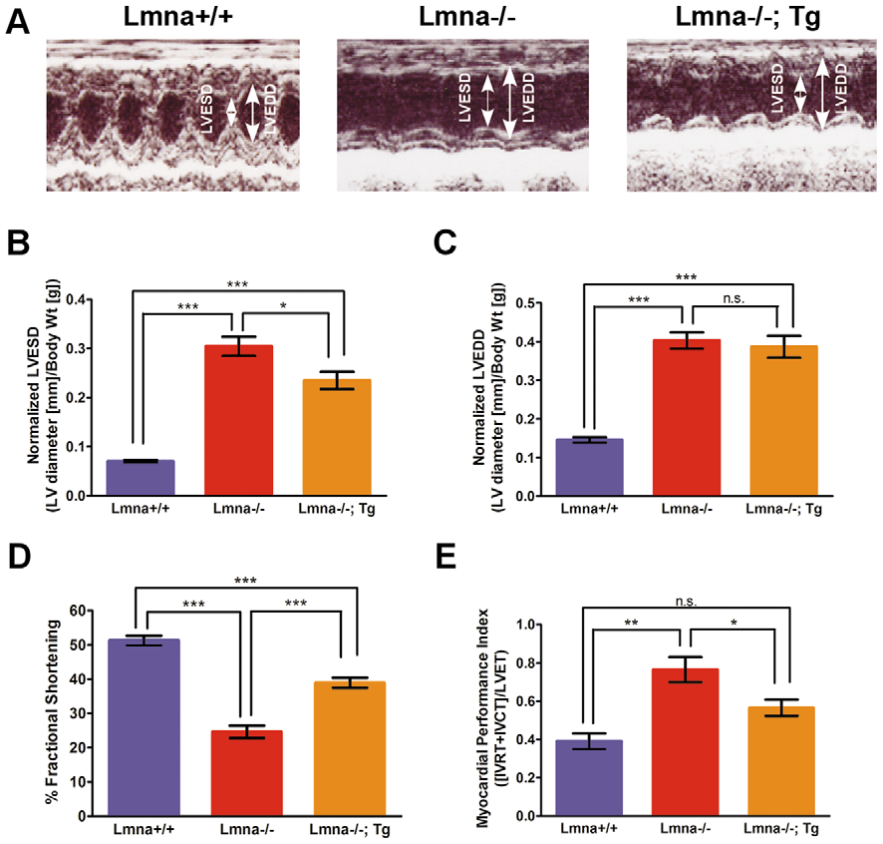
*Lmna* ^−/−^; Tg mice display improved contractile function compared to *Lmna* ^−/−^ littermates. (A) Representative echocardiograms of *Lmna* ^−/−^, *Lmna* ^−/−^; Tg, and control littermates. (B) Normalized left ventricular end-systolic diameter (LVESD) measurements are increased in *Lmna* ^−/−^ hearts compared to control hearts and are improved in *Lmna* ^−/−^; Tg hearts. (C) Normalized left ventricular end-diastolic diameter (LVEDD) measurements are increased in *Lmna* ^−/−^ hearts and are not improved in *Lmna* ^−/−^; Tg hearts. (D) Fractional shortening is decreased in *Lmna* ^−/−^ hearts compared to control hearts and is improved in *Lmna* ^−/−^; Tg hearts. (E) Myocardial performance index (MPI) is increased (worse) in *Lmna* ^−/−^ mice, but is improved in *Lmna* ^−/−^; Tg mice. For all of the above experiments: Control, N = 13; *Lmna* ^−/−^, N = 15; *Lmna* ^−/−^; Tg, N = 12 for 4–8 weeks of age. One-way ANOVA analyses were performed (B–E) and significant genotype differences are listed above for each panel. Post-tests were performed between genotypes and significance is listed as follows: * P<0.05; ** P<0.01; *** P<0.001; n.s. not significant.

To determine whether transgenic expression of FLAG-lamin A improves cardiac function in *Lmna* ^−/−^ mice, transthoracic echocardiograms were performed at 4–8 weeks of age. Echocardiographic measurements comparing *Lmna* ^+/+^ and *Lmna* ^+/+^; Tg mice showed no significant difference **(Figure S1 A–C)**. *Lmna* ^−/−^ mice display impaired contractility and left ventricular dilatation **(** **Figure 3A** **)**. They have significantly increased (P<0.001) left ventricular end-systolic diameters (LVESD) and left ventricular end-diastolic diameters (LVEDD) normalized for body weight between *Lmna* ^−/−^ and *Lmna* ^+/+^ littermates **(** **Figures 3B & 3C** **)**. Additionally, *Lmna* ^−/−^ hearts are enlarged as shown by the ∼55% increase in the left ventricular mass index (LVMI) **(Figure S2)**. Fractional shortening decreased by 50% in *Lmna* ^−/−^ mice compared to *Lmna* ^+/+^ littermates (P<0.001) **(** **Figure 3D** **)** and consistent with previous observations [8]. As a second measure of cardiac function, the myocardial performance index (MPI) was calculated (See methods for further details; increased MPI scores have been shown previously to reflect LV systolic and/or diastolic dysfunction [18]). *Lmna* ^−/−^ mice display 100% increase in MPI compared to *Lmna* ^+/+^ littermates (P<0.001) **(** **Figure 3E** **)**, consistent with decreased cardiac contractility in *Lmna* ^−/−^ mice.

The expression of FLAG-lamin A partially restores cardiac contractility in *Lmna* ^−/−^ mice **(** **Figures 3A, 3B, 3D** **)**. Compared to *Lmna* ^−/−^ mice, *Lmna* ^−/−^; Tg mice significantly attenuate dilation of LVESD by ∼25% (P<0.01) **(** **Figure 3B** **)** and display ∼60% improvement in fractional shortening (P<0.001) although fractional shortening is still 20% lower in comparison to control littermates (P<0.001) **(** **Figure 3D** **)**. Similarly, the MPI is partially improved by ∼25% in *Lmna* ^−/−^; Tg mice compared to *Lmna* ^−/−^ mice (P<0.01) **(** **Figure 3E** **).** However, hearts of *Lmna* ^−/−^; Tg mice still exhibit cardiac enlargement, as LVEDD **(** **Figure 3C** **)** and LVMI **(Figure S2)** are not significantly changed compared to hearts of *Lmna* ^−/−^ mice. Furthermore, mRNA expression of atrial natriuretic factor and brain natriuretic peptide, hallmarks of cardiac remodeling and physical stress, are not significantly changed in hearts of *Lmna* ^−/−^; Tg mice compared to those from *Lmna* ^−/−^ mice **(Figure S3)**. Collectively, these data indicate that the mosaic expression of FLAG-lamin A in *Lmna* ^−/−^ cardiomyocytes results in partial but significant restoration of cardiac contractility compared to *Lmna* ^−/−^, yet fails to ameliorate cardiac dilation and remodeling.

### Characterization of molecular phenotypes associated with cardiac conduction in both *Lmna* ^−/−^ and *Lmna* ^−/−^; Tg mice

**Figure 4:**
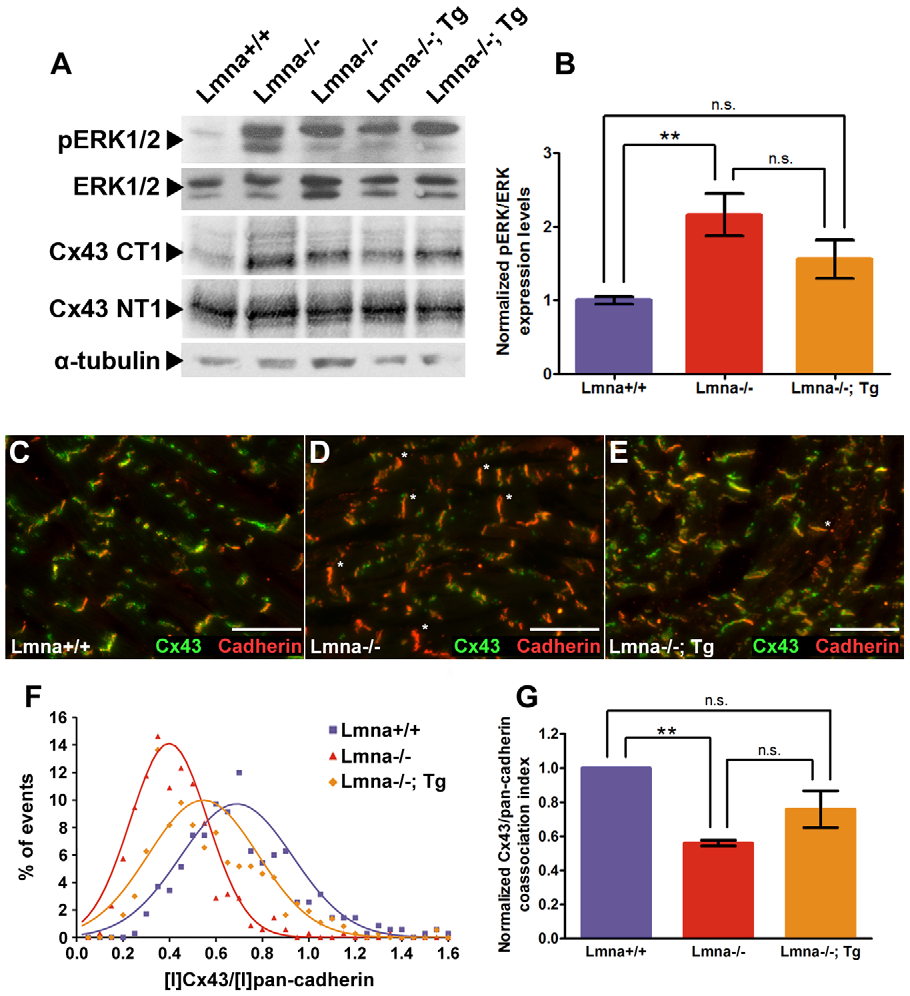
Transgenic expression of lamin A in *Lmna* ^−/−^ cardiomyocytes partially restores ERK1/2 activation and gap junction protein localization. (A) Western blot analysis of *Lmna* ^+/+^, *Lmna* ^−/−^, and *Lmna* ^−/−^; Tg mouse heart lysates. Phosphorylated-ERK1/2 (pERK1/2) levels are increased in *Lmna* ^−/−^ compared to *Lmna* ^+/+^ and are decreased in *Lmna* ^−/−^; Tg hearts while total ERK1/2 levels are unchanged. Total Cx43 protein levels detected by NT1 are unchanged, while cytoplasmic levels of Cx43 as detected by CT1 are increased. α-tubulin is shown as a loading control. (B) Quantification of pERK1/2 levels normalized to total ERK1/2 levels in 5–7 week old *Lmna* ^+/+^ (N = 13), *Lmna* ^−/−^ (N = 16), and *Lmna* ^−/−^; Tg (N = 11) mouse heart lysates. One-way ANOVA analyses were performed for significance with post-test significance values as follows: ** P<0.01; n.s. not significant. (C–E) Indirect immunofluorescence micrographs of pan-cadherin (red) and Cx43 (green) in *Lmna* ^+/+^ (C), *Lmna* ^−/−^ (D), and (E) *Lmna* ^−/−^; Tg heart ventricle sections taken at equivalent exposure times from 6-week old mice. Asterisks mark intercalated discs with qualitative decreases in Cx43 relative to pan-cadherin staining. White scale bar denotes 50 µm. (F) Distribution of individual intercalated disks summarizing relative frequency of Cx43 co-association index in 5–7-week old *Lmna* ^+/+^ (N = 3; 350 intercalated discs analyzed total), *Lmna* ^−/−^ (N = 3; 349 intercalated discs analyzed total), and *Lmna* ^−/−^; Tg (N = 3; 367 intercalated discs analyzed total) mice. A non-linear regression for Gaussian distribution was fitted for each group tested (*Lmna* ^+/+^ R^2^ = 0.91; *Lmna* ^−/−^ R^2^ = 0.96; *Lmna* ^−/−^; Tg R^2^ = 0.80). (G) Combined Cx43 co-association indices from multiple experiments normalized against their respective *Lmna* ^+/+^ control (N = 3). One-way ANOVA analyses were performed for significance with post-test significance values as follows: ** P<0.01; n.s. not significant.

Increased ERK signaling as reflected by levels of phosphorylated ERK1/2 (pERK1/2) has been observed in hearts of *Lmna^H222P/H222P^* mice [19] and in other cell systems with knockdown of A-type lamin or emerin expression [20]. Consistently, we detect a 2.5-fold increase of pERK1/2 in *Lmna* ^−/−^ hearts relative to total ERK1/2 levels (P<0.01) **(** **Figure 4A and 4B** **)**. Although we were unable to achieve significance, *Lmna* ^−/−^; Tg (n = 11) hearts trend towards a reduced pERK1/2 level compared to hearts from *Lmna* ^−/−^ mice (n = 16), suggesting that there may be a partially decreased cellular stress response. Increases in ERK1/2 activity have been shown to negatively affect gap junction communication through phosphorylation of connexin43 (Cx43) [21], [22]. We find no significant change in levels of total Cx43 as detected by the NT1 antibody. However, in *Lmna* ^−/−^ hearts we detect a significant increase in CT1 signal, an antibody which predominantly recognizes Cx43 found in the cytoplasm and is increased in ischemic hearts [23] **(** **Figure 4A** **)**. The increase in CT1 signal is not significantly attenuated in the hearts from *Lmna* ^−/−^; Tg mice. In addition to changes detectable by immunoblot, we also observe a decreased amount of gap junctional Cx43 in hearts from 5–7 week old *Lmna* ^−/−^ mice compared to similarly aged *Lmna* ^+/+^ mice via immunofluorescence **(** **Figures 4C and 4D** **)**. Hearts from *Lmna* ^−/−^; Tg mice appear to have more Cx43 present at the gap junction than hearts from *Lmna* ^−/−^ mice **(** **Figure 4E** **)**. To quantitatively assay the loss of Cx43 in the intercalated discs of ventricular myocytes from 5–7 week old *Lmna* ^−/−^ mice, we formulated a co-association index for scoring Cx43 at intercalated discs (see methods). Intercalated discs from *Lmna* ^+/+^ ventricular myocytes display a mean Cx43 index of 0.70, which is reduced to 0.39 in *Lmna* ^−/−^ ventricular myocytes **(** **Figure 4F** **)**. Intercalated discs from *Lmna* ^−/−^; Tg ventricular myocytes display a mean Cx43 index of 0.53. When normalized against the mean index for *Lmna^+/+^* ventricular myocytes for each experiment, ventricular myocytes from *Lmna* ^−/−^ mice display 45% decrease in Cx43 localization at the intercalated disc (P<0.01) **(** **Figure 4G** **)**. In contrast, ventricular myocytes from *Lmna* ^−/−^; Tg mice display 25% decrease in Cx43 localization at the intercalated disc compared to *Lmna^+/+^* ventricular myocytes (P = 0.11), suggesting a partial restoration of Cx43 localization at the intercalated disc.

### Transgenic *Lmna* ^−/−^ mice have less severe conduction defects

**Figure 5:**
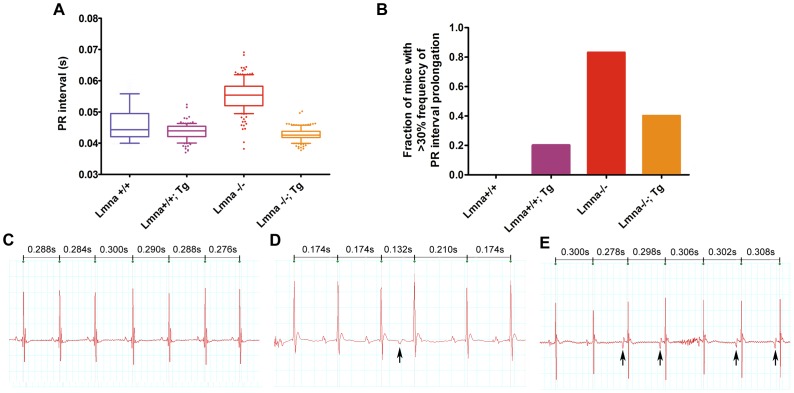
Lmna^−/−^; Tg mice show improved conduction parameters compared to Lmna^−/−^ mice by ECG analysis. (A) Box-and-whiskers plot of pooled *Lmna* ^+/+^ PR intervals (N = 5) compared with single animal PR intervals for each of the following genetic backgrounds at 5-7 weeks of age: *Lmna* ^+/+^; Tg, *Lmna* ^−/−^ and *Lmna* ^−/−^; Tg. The whiskers represent at 5–95% confidence interval and individual outliers are represented by their respective dots. (B) Fraction of mice which display >30% abnormally prolonged PR interval. *Lmna* ^+/+^ (N = 5), *Lmna* ^+/+^; Tg (N = 5), *Lmna* ^−/−^ (N = 6), and *Lmna* ^−/−^; Tg (N = 5). (C–E) ECG traces from a *Lmna* ^+/+^ mouse (C), a *Lmna* ^−/−^ mouse exhibiting an atrial premature beat (D) and a *Lmna* ^−/−^ mouse displaying multifocal atrial rhythm (E). Note the changes in P-wave morphology and cycle length. Arrows denote ectopic P-waves. RR intervals are shown above.

We hypothesized that if Cx43 localization was partially restored in *Lmna* ^−/−^; Tg mice, there may be improvements in cardiac conduction. To test this hypothesis, we took electrocardiography (ECG) recordings of 5–7 week old mice and measured a number of parameters associated with cardiac conduction, including PR and QRS intervals. Despite the previous study reporting a significant increase in the average PR and QRS intervals for *Lmna* ^−/−^ mice [8], we were unable to observe a significant increase in the average PR or QRS intervals of similarly aged *Lmna* ^−/−^ mice after analyzing 300 beats per mouse (N = 5) using this method **(Table S1)**. However, intermittent prolongation of the PR interval was noted in *Lmna* ^−/−^ mice. Since there is no consensus on the normal reference values for PR intervals, we derived the reference values by analyzing 1,500 beats from 5 *Lmna^+/+^* littermates, which display a Gaussian distribution. PR intervals from single mice were then compared against the derived reference value and values greater than 95% of our normal reference were considered abnormally prolonged **(** **Figure 5A** **)**. Using the cut-off value of >30% abnormally prolonged PR intervals, 5 out of 6 *Lmna* ^−/−^ mice displayed PR prolongation (Fisher's exact test, P = 0.0476), suggesting the presence of first degree atrioventricular block **(** **Figure 5B** **)**. In contrast, 2 out of 5 *Lmna* ^−/−^; Tg mice exhibited PR prolongation (Fisher's exact test, P = 0.2222), suggesting partial improvement in atrioventricular conduction due to cardiac-specific FLAG-lamin A expression. One *Lmna* ^+/+^; Tg mouse exhibited >30% PR interval prolongation and a second mouse displayed a dropped beat **(Figure S4)**, which might be a manifestation of sinus pause, sinoatrial or atrioventricular block, suggesting that increased expression of lamin A in a subset of normal myocytes can also result in conduction abnormalities. We also observe several arrhythmic events in *Lmna* ^−/−^ mice. Compared to a normal ECG tracing from a representative *Lmna* ^+/+^ mouse **(** **Figure 5C** **)**, the ECG of *Lmna* ^−/−^ mice display intermittent atrial premature beat **(** **Figure 5D** **)** and a multifocal atrial rhythm **(** **Figure 5E** **)**. We were unable to locate any similar arrhythmic occurrences in *Lmna* ^−/−^; Tg mice, potentially due to improved cardiac function.

### Transgene expression of FLAG-lamin A in *Lmna* ^−/−^ cardiomyocytes extends lifespan

**Figure 6:**
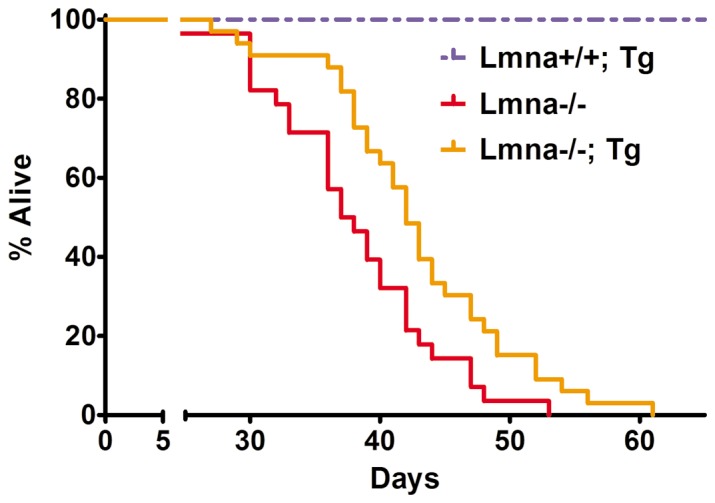
Extension of median and maximal lifespan in *Lmna* ^−/−^ mice expressing cardiac lamin A. *Lmna* ^−/−^; Tg mice display a 12% and 15% increase in median and maximal lifespans, respectively, compared to *Lmna* ^−/−^ littermates (*Lmna* ^+/+^; Tg N = 10; *Lmna* ^−/−^ N = 24; *Lmna* ^−/−^; Tg N = 28). The Log-Rank test, which measures significance evenly across all time points, reports a significance probability of P<0.0114

Concurrent with our studies of cardiac function and molecular restoration in cardiomyocytes from *Lmna* ^−/−^; Tg mice, we sought to determine whether this improvement would translate into an increased lifespan. Kaplan-Meier curves were generated from a cohort of 24 and 28 mice each for transgenic and non-transgenic *Lmna* ^−/−^ mice, respectively. Despite the mosaic expression of FLAG-lamin A in *Lmna* ^−/−^; Tg cardiomyocytes, we observe a 12% mean increase and a 15% maximal increase in lifespan (P<0.011) of *Lmna* ^−/−^; Tg mice compared to non-transgenic *Lmna* ^−/−^ littermates **(** **Figure 6** **)**.

## Discussion

In this study, we tested the hypothesis that cardiomyocyte-specific expression of lamin A in *Lmna* ^−/−^ mice can restore cardiac function and increase lifespan. Through the generation of *Lmna* ^−/−^ mice with cardiomyocyte-specific expression of FLAG-lamin A, we observed significantly improved fractional shortening and myocardial performance index by echocardiogram, restored localization of both desmin and Cx43, and attenuated protein levels of both pERK1/2 and desmin, resulting in partial restoration of cardiac function compared to *Lmna* ^−/−^ mice. Despite increased cardiac contractility, cardiac remodeling in transgenic *Lmna* ^−/−^ mice was still evident with no amelioration of chamber dilation. We observed less Cx43 localized to the intercalated disk in ventricular myocytes of *Lmna* ^−/−^ mice which was partially restored in ventricular myocytes of *Lmna* ^−/−^; Tg mice. This modest improvement in Cx43 localization was also consistent with our finding that the stochastic PR interval prolongation observed in *Lmna* ^−/−^ mice is less frequent in *Lmna* ^−/−^; Tg mice. The improvements to cardiac function due to the expression of lamin A resulted in a significant—though modest—extension in lifespan compared to *Lmna* ^−/−^ littermates. Collectively, these data suggest that cardiomyocyte-specific expression of lamin A in *Lmna* ^−/−^ mice can partially rescue cardiac function and that the cardiac pathology present in *Lmna* ^−/−^ mice is lifespan limiting.

Mosaic expression of lamin A in *Lmna* ^−/−^ cardiomyocytes was very likely a limiting factor in many of our incompletely rescued phenotypes, but also allowed us to observe a juxtaposition of cardiomyocytes either expressing or not expressing the lamin A transgene. Other studies had previously used this approach to address cell autonomy roles in the cardiac system [24], [25]. In our study, *Lmna* ^−/−^; Tg mice displayed ∼30–40% heterogeneity of lamin A transgene expression in ventricular cardiomyocytes, and we observed both cell-autonomous and non-cell-autonomous phenotypes. In general, our findings suggest the contractile defect of *Lmna* ^−/−^ mice can be rescued in a cell-autonomous fashion, as indicated by restoration of the desmin cytoskeletal network in lamin A-expressing *Lmna* ^−/−^ cardiomyocytes. We also observed a significantly decreased fraction of non-transgene expressing *Lmna* ^−/−^; Tg cardiomyocytes with disorganized desmin, which we postulate as being propagated from neighboring cardiomyocytes expressing the lamin A transgene. Since mechanotransduction relies on communication between the nucleus and extracellular interactions from cell-cell contact, extracellular matrix composition, and secreted factors [26], [27], we propose that A-type lamins may play an additional role by modulating these extracellular properties to coordinate mechanosensing and transduction in a non-cell-autonomous manner.

Altered A-type lamin function also results in increased activity of ERK1/2. Although this phenomenon is universal to many cell types with abnormal A-type lamin composition [20], [28], [29], [30], the administration of the MEK inhibitor, PD98059, improves the dilated cardiomyopathy of *Lmna^H222P/H222P^* mice [31], [32], which strongly supports the notion that altered ERK1/2 activity is a critical component associated with pathogenesis. Indeed, increased ERK1/2 activity is associated with cardiac hypertrophy in other heart disease models [33]. Cx43 is the most widely distributed member of the connexin family of proteins, which forms gap junctions, facilitates cell-to-cell communication, and is found in a variety of different tissues and cell types [34]. Phosphorylation of Cx43 by ERK1/2 inhibits gap-junctional communication [21], [22], and decreased Cx43 activity at the intercalated disc in *Lmna* ^−/−^ mice may play a critical part in the conduction defects and premature death observed. Electrical conduction of the heart cannot be completely rescued in a cell-autonomous fashion as gap junctions must form stable connexons with neighboring cells in order to maintain a functioning channel, making it an attractive example of non-cell-autonomous function. Mice carrying a heterozygous deletion for Cx43 [35], as well as cardiac-restricted inactivation of Cx43 in adult mice [36], [37], show slowing of ventricular conduction and eventual death by ventricular tachycardia. In addition, heterogeneous or mosaic expression of Cx43 resulted in similar spontaneous ventricular arrhythmia and altered conduction velocity [25], [38]. We observed heterogeneity in the rescue of gap-junctional Cx43 levels, which we speculate contributes to the continued premature death phenotype of *Lmna* ^−/−^; Tg mice through terminal arrhythmic events. Interestingly, a similar phenotype describing loss of Cx43 localization to the intercalated disc coupled with desmin aggregation has been described in D7-des mice, which encode a deletion that causes human dilated cardiomyopathy [39]. Additionally, we observed arrhythmia in two *Lmna* ^+/+^; Tg mice suggesting that increased expression of lamin A in a fraction of cardiomyocytes can alter conduction—and possibly connexin activity—in an otherwise normal heart. These examples demonstrate the importance of assessing non-cell-autonomous outcomes in any transgenic and gene therapy models.

ERK1/2 activity can also result in the activation of different transcriptional pathways depending on the context of the tissue type and origin of ERK1/2 activation [40]. A-type lamins can bind and sequester c-Fos, the immediate downstream effector of pERK1/2, thereby inhibiting the activity of AP-1 [41]. Further studies have shown that active ERK1/2 can also co-localize with lamin A and c-Fos at the nuclear envelope, and the loss of A-type lamins results in increased AP-1 activity and reduced c-Fos phosphorylation [29]. Finally, ERK1/2 may additionally act upon Cx43 in a cell survival pathway, as ERK1/2 activity enhances translocation of Cx43 to the mitochondria, where Cx43 has been shown to play a protective role against cell death [42], [43], [44]. Although it is currently not known whether ERK1/2 signaling can modulate desmin filament formation in cardiomyocytes, it is becoming increasingly clear that loss of or mutation of A-type lamins may impinge on multiple pathways that lead to cardiac dysfunction.

Our data highlight the role of A-type lamins in cardiomyocyte function to both maintain efficient contraction and preserve a functional conductive network. These findings also re-emphasize the need for uniform expression in gene therapy models correcting conduction diseases.

## Methods

### Animals

*Lmna* ^+/−^ mice were obtained from Dr. Colin Stewart [7] and were backcrossed on C57BL/6J for 9 additional generations. The resulting *Lmna* ^+/−^ mice, B6.129S1(Cg)-Lmna^tm1Stw^/BkknJ, were crossed with transgenic mice (founder line 903; B6/CBA F1 hybrids) expressing FLAG-lamin A under the control of the α-myosin heavy chain promoter, which have been described elsewhere [16]. The resulting transgenic *Lmna* ^+/−^ mice were crossed with *Lmna* ^+/−^ mice to generate *Lmna* ^−/−^ mice expressing the cardiac-specific transgene (*Lmna* ^−/−^; Tg). Details for genotyping can be found in **Methods S1**. Mice were bred and maintained under specific pathogen-free conditions. All experiments were performed in compliance with the University of Washington Institutional Animal Care and Use Committee (Protocol Number: 2174–23).

### Tissue preparation and indirect immunofluorescence

Hearts of transgenic and non-transgenic *Lmna* ^−/−^ and control mice were rapidly excised and rinsed in phosphate-buffered saline (PBS) prior to mounting in Tissue-Tek O.C.T. compound and subsequent freezing in liquid nitrogen-cooled isopentane. Heart ventricle sections 8 µm in thickness were collected and mounted on Superfrost plus glass slides (Fisher). Antibodies used and detailed methods for staining and scoring desmin and connexin can be found in **Methods S1**.

### Western analysis

Tissues were homogenized using the TH homogenizer (Omni International) in a lysis buffer (10 µL/mg tissue) containing the following: 2% SDS, 250 mM sucrose, 75 mM urea, 1 mM DTT, 50 mM Tris (pH 7.5), 25 µg/mL aprotinin, and 10 µg/mL leupeptin [13], [19]. Antibodies used and further details can be found in **Methods S1**.

### Echocardiography

Transgenic and non-transgenic *Lmna* ^−/−^ mice and control littermates received echocardiography examination (Siemens Acuson CV70). A mixture of 0.5% isoflurane with O_2_ was administered through a nose cone to provide adequate sedation but minimal cardiac suppression during echocardiography. Heart rate was monitored and kept above 400 bpm to minimize any effect on any parameters [45]. Pathologists performing the echocardiography examination were blinded to the transgene genotype for *Lmna* ^−/−^ mice. Further details can be found in **Methods S1**.

### ECG recordings

Electrocardiography was recorded using TA11ETA-F10 transmitters (Data Science International, St.Paul, MN). ECG data were recorded using Dataquest A.R.T™software (Data Sciences International) and the first 300 beats were analyzed using LabChart7 Pro (ADInstruments). Further details can be found in **Methods S1**.

### Statistical analysis

Data are expressed as mean ± S.E.M. and one-way ANOVA analyses were performed where relevant with Bonferroni post tests to compare genotypes of specific age groups. A value of P<0.05 was considered statistically significant. For lifespan analysis, a Kaplan-Meier curve was generated and Log Rank Tests were performed with a value of P<0.05 that was considered statistically significant. All statistical analyses and graphs were performed using GraphPad Prism 5.02.

## Supporting Information

Figure S1 ***Lmna*** **^+/+^ and** ***Lmna*** **^+/+^; Tg hearts show no significant difference in cardiac function.** (A & B) No significant difference is noted in (A) fractional shortening, (B) myocardial performance index (MPI), or (C) left ventricular mass index (LVMI) of *Lmna* ^+/+^ and *Lmna* ^+/+^; Tg hearts at 4–8 weeks of age as measured by echocardiography. Two-tailed unpaired t-tests were used to determine P-values which are listed for each panel. (*Lmna* ^+/+^, N = 6; *Lmna* ^+/+^; Tg, N = 7).
(TIF)

Figure S2 ***Lmna^−/−^*** **hearts are enlarged relative to control littermates and** ***Lmna^−/−^*** **; Tg hearts are not significantly improved.** Left ventricular mass of 4–8 week old mice was measured and normalized to body weight to resolve the LVMI. One-way ANOVA was performed and significant genotype differences are listed for each panel. Bonferonni post-tests were performed between genotypes and significance is listed as follows: * P<0.05; n.s. not significant. (Control, N = 13; *Lmna^−/−^*, N = 15; *Lmna^−/−^*; Tg, N = 12).
(TIF)

Figure S3 **mRNA levels of global remodeling markers, ANF and BNP, are enriched in** ***Lmna*** **^−/−^ and** ***Lmna*** **^−/−^; Tg mice.** qPCR of cardiac remodeling mRNA's for *Lmna* ^−/−^ and *Lmna* ^−/−^; Tg hearts. Data are presented as fold-increase over *Lmna* ^+/+^ hearts. Global cardiac remodeling mRNA's, ANF and BNP, are all increased in *Lmna* ^−/−^ hearts and are not significantly changed in *Lmna* ^−/−^; Tg hearts. (*Lmna^+/+^*, N = 4; *Lmna* ^−/−^, N = 7; *Lmna* ^−/−^; Tg, N = 5).
(TIF)

Figure S4 **Dropped beat in** ***Lmna*** **^+/+^; Tg heart.** An isolated case of a dropped heartbeat was noted in a single *Lmna* ^+/+^; Tg heart during ECG recording which could reflect either a sinus pause or sino-atrial block.
(TIF)

Table S1 **Average ECG parameters from 5–7 week old mice.** ECG parameters of individual mice from *Lmna^+/+^* and *Lmna* ^−/−^ mice either expressing or not expressing FLAG-lamin A in cardiomyocytes. Similar mouse genotypes are grouped together with mouse ID displayed and parameters are averaged. Each parameter from an individual mouse represents an averaged value from the first 300 beats recorded. Parameters include RR interval, Heart Rate, PR interval, P duration, and QRS interval.
(TIF)

Methods S1 **Additional information and specifics on methods with supporting references.** Further details include genotyping primers, qPCR primers, specific antibodies used for both immunofluorescence and Western blotting, details on image analysis, and animal techniques.
(DOC)
