# Correction: Cardiomyocyte-Specific Expression of Lamin A Improves Cardiac Function in *Lmna* ^−/−^ Mice

**DOI:** 10.1371/annotation/92be6b32-d8e7-44c2-80a9-21097ad27965

**Published:** 2012-09-17

**Authors:** Richard L. Frock, Steven C. Chen, Dao-Fu Dai, Ellie Frett, Carmen Lau, Christina Brown, Diana N. Pak, Yuexia Wang, Antoine Muchir, Howard J. Worman, Luis F. Santana, Warren C. Ladiges, Peter S. Rabinovitch, Brian K. Kennedy

The name of the third author was misspelled.

The correct name is: Dao-Fu Dai.

The correct citation is: Frock RL, Chen SC, Dai D-F, Frett E, Lau C, et al. (2012) Cardiomyocyte-Specific Expression of Lamin A Improves Cardiac Function in Lmna−/− Mice. PLoS ONE 7(8): e42918. doi:10.1371/journal.pone.0042918

In addition, the first two authors, Richard L. Frock and Steven C. Chen, should be noted as contributing equally to this work. 

