# Supplementary figures and images for: Cardiomyocyte-Specific Expression of Lamin A Improves Cardiac Function in Lmna −/− Mice
Source: PLoS One. 2012 Aug 15;7(8):e42918. doi: 10.1371/journal.pone.0042918 (PMC3419749; doi:10.1371/journal.pone.0042918)

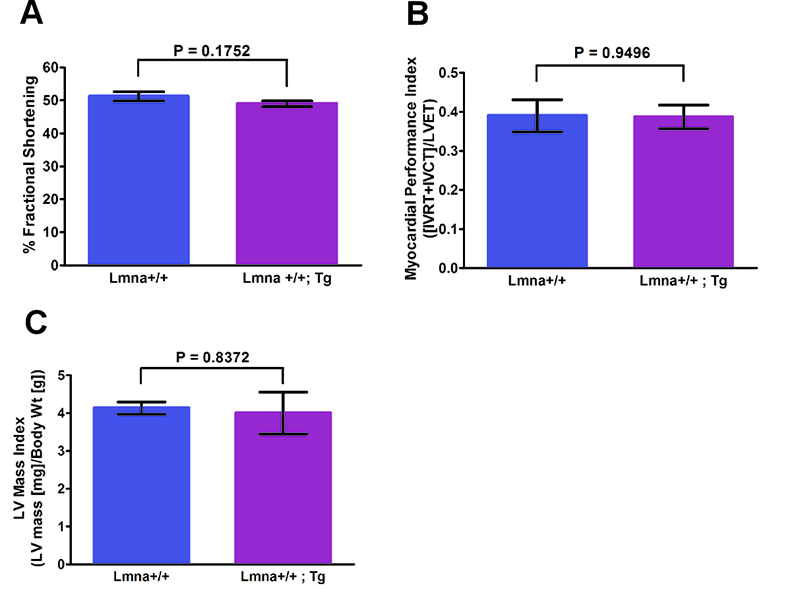

Supplement: Figure S1 — Lmna +/+ and Lmna +/+; Tg hearts show no significant difference in cardiac function. (A & B) No significant difference is noted in (A) fractional shortening, (B) myocardial performance index (MPI), or (C) left ventricular mass index (LVMI) of Lmna +/+ and Lmna +/+; Tg hearts at 4–8 weeks of age as measured by echocardiography. Two-tailed unpaired t-tests were used to determine P-values which are listed for each panel. (Lmna +/+, N = 6; Lmna +/+; Tg, N = 7). (TIF) [file pone.0042918.s001.tif]

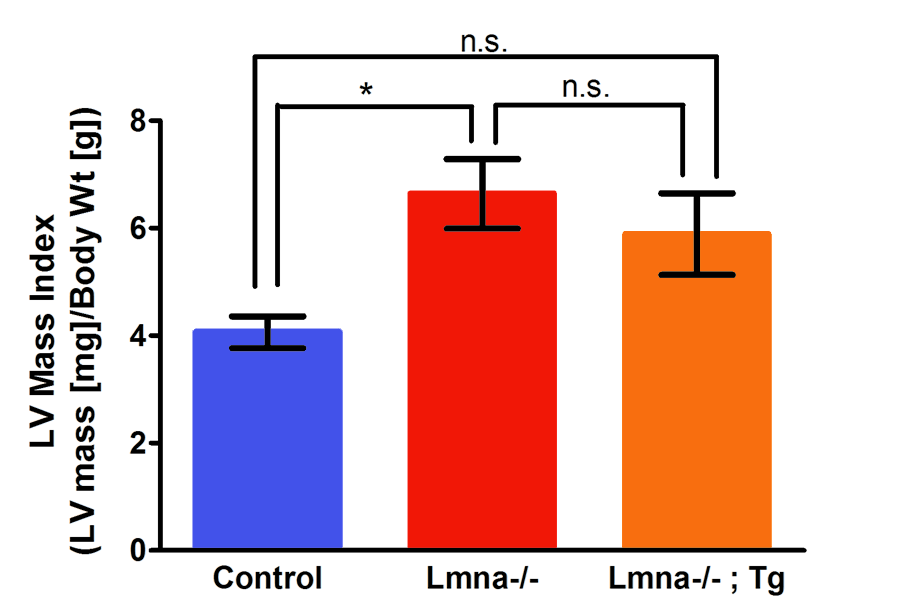

Supplement: Figure S2 — Lmna−/− hearts are enlarged relative to control littermates and Lmna−/− ; Tg hearts are not significantly improved. Left ventricular mass of 4–8 week old mice was measured and normalized to body weight to resolve the LVMI. One-way ANOVA was performed and significant genotype differences are listed for each panel. Bonferonni post-tests were performed between genotypes and significance is listed as follows: * P<0.05; n.s. not significant. (Control, N = 13; Lmna−/−, N = 15; Lmna−/−; Tg, N = 12). (TIF) [file pone.0042918.s002.tif]

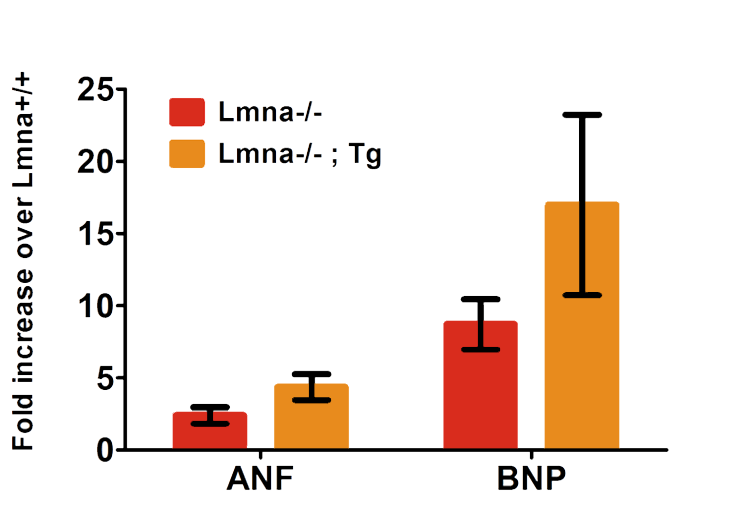

Supplement: Figure S3 — mRNA levels of global remodeling markers, ANF and BNP, are enriched in Lmna −/− and Lmna −/−; Tg mice. qPCR of cardiac remodeling mRNA's for Lmna −/− and Lmna −/−; Tg hearts. Data are presented as fold-increase over Lmna +/+ hearts. Global cardiac remodeling mRNA's, ANF and BNP, are all increased in Lmna −/− hearts and are not significantly changed in Lmna −/−; Tg hearts. (Lmna+/+, N = 4; Lmna −/−, N = 7; Lmna −/−; Tg, N = 5). (TIF) [file pone.0042918.s003.tif]

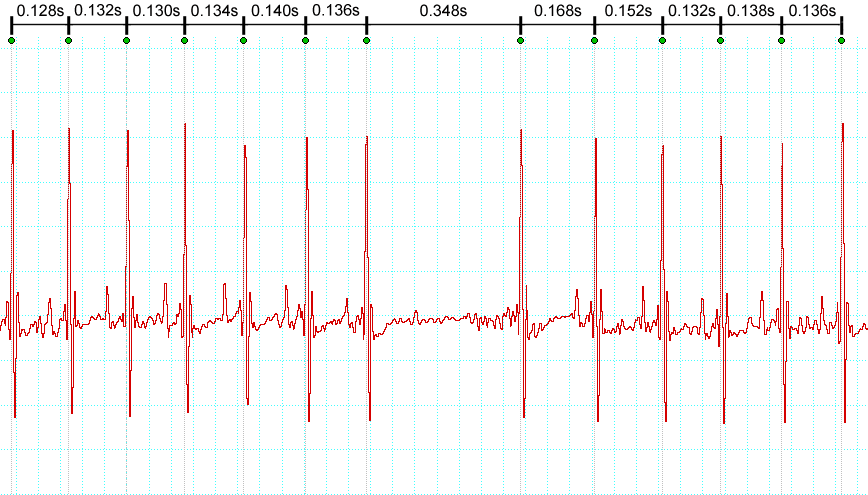

Supplement: Figure S4 — Dropped beat in Lmna +/+; Tg heart. An isolated case of a dropped heartbeat was noted in a single Lmna +/+; Tg heart during ECG recording which could reflect either a sinus pause or sino-atrial block. (TIF) [file pone.0042918.s004.tif]

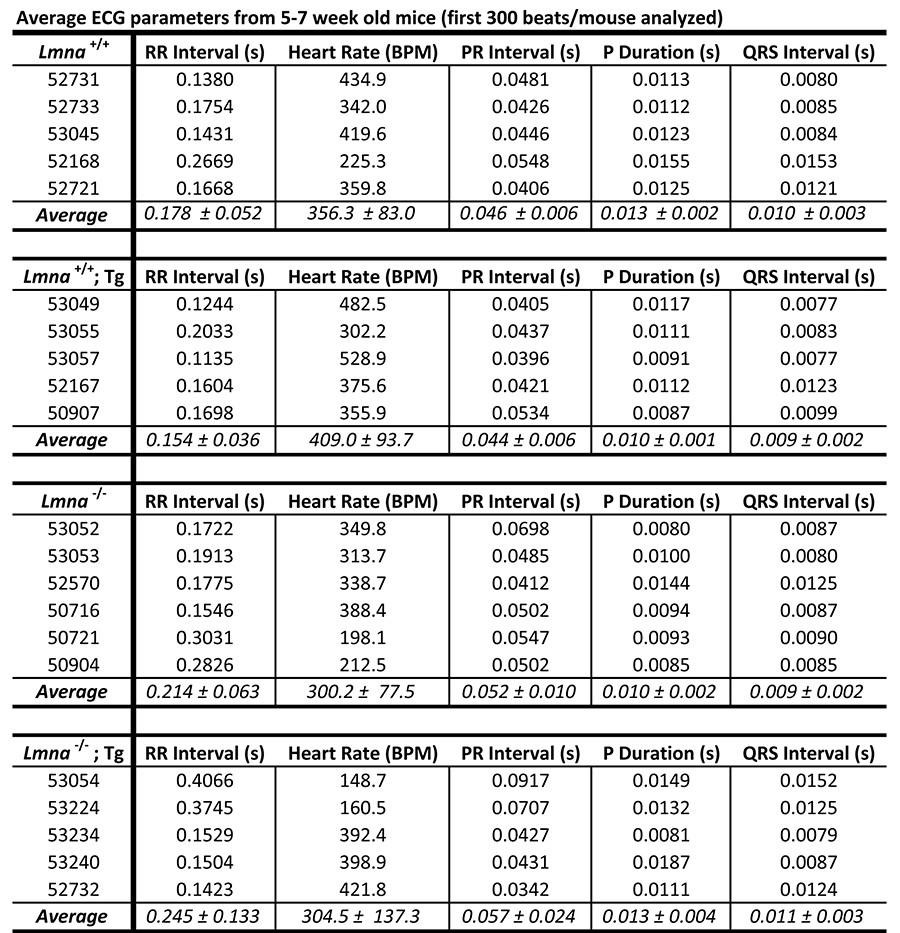

Supplement: Table S1 — Average ECG parameters from 5–7 week old mice. ECG parameters of individual mice from Lmna+/+ and Lmna −/− mice either expressing or not expressing FLAG-lamin A in cardiomyocytes. Similar mouse genotypes are grouped together with mouse ID displayed and parameters are averaged. Each parameter from an individual mouse represents an averaged value from the first 300 beats recorded. Parameters include RR interval, Heart Rate, PR interval, P duration, and QRS interval. (TIF) [file pone.0042918.s005.tif]
