# Supplementary material for: Cardiomyocyte-Specific Expression of Lamin A Improves Cardiac Function in Lmna −/− Mice
Source: PLoS One. 2012 Aug 15;7(8):e42918. doi: 10.1371/journal.pone.0042918 (PMC3419749; doi:10.1371/journal.pone.0042918)
Supplement: Methods S1 — Additional information and specifics on methods with supporting references. Further details include genotyping primers, qPCR primers, specific antibodies used for both immunofluorescence and Western blotting, details on image analysis, and animal techniques. (DOC) [file pone.0042918.s006.doc]

**Supplemental Methods**

***Animals***

Genotyping of transgenic mice was performed by using PCR using primers against the FLAG-lamin A transgene: FLAG – ATGGACTACAAGGACGACGATGAC; Lamin A – AGTTCAGCAGAGCCTCCAGGTCCTT yielding a ~500 bp fragment. Primers for the detection of *Lmna* and its knockout allele : 5’-wild-type – TGCTGATGCCATGGATACTC; 5’-knockout – GCACGAGACTAGTGAGACGTG; 3’-common – GAGAAGGCAGAGGTGTGAGCAGC yielding ~1 kb and 700 bp fragments respectively.

***Tissue preparation and indirect immunofluorescence***

Primary antibodies were myosin heavy chain (DSHB MF20; 1:100), desmin (Abcam 15200; 1:250), pan-cadherin (Sigma CH-19; 1:200), FLAG (Sigma M2; 1:50), and connexin 43 (Invitrogen 71-0700; 1:200). Sections were fixed in cold acetone for 20 min at –20°C, washed in PBS prior to blocking in 20% goat serum, 0.1% BSA, PBS for 20 min for desmin and FLAG staining. Blocking with 1% BSA, 0.3% TritonX-100 in PBS for 15 minutes was done for staining of pan-cadherin and connexin 43.

For nuclear staining of the FLAG-tagged transgenic protein, sections were boiled for 10 minutes in antigen unmasking solution (Vector labs) prior to blocking. Samples were viewed on a Zeiss 200M Axiovert and images were acquired using Axiovision. Scoring of desmin phenotypes from collected images was performed using ImageJ (NIH). Scoring of desmin accumulation was accomplished using tools from ImageJ to differentially mark fibers with qualitatively increased desmin staining in the cytoplasm or loss of normal striated pattern (include total number here if not apparent in the legend) compared to control animal hearts. The co-association index was determined by measuring the ratio of fluorescence intensities of connexin 43 overlapping with pan-cadherin at the intercalated discs of sectioned ventricular myocytes using the "Outline Spline" tool to trace intercalated discs and then calculated the “Densitometric Mean” for each channel utilizing Axiovision software. Images were taken for each group at equivalent exposure times for connexin 43 and pan-cadherin. Fluorescence intensity ratios were binned into 0.05 increments from 0-1.0, where 1.0 represents equal intensity of connexin 43 with pan-cadherin. A non-linear regression for Gaussian distribution was applied to each group to determine mean co-association indices per experiment. To compare multiple experiments, mean co-association indices for each experiment were normalized to their respective mean index for *Lmna*^+/+^ and averages of the normalized means were graphed.

***Western analysis***

Protein concentrations of lysates were quantitated using a DCA assay kit (Biorad). Approximately 10 μg of total protein was loaded for detection of desmin protein and 50 μg of total protein was loaded for detection of other less abundant proteins. Protein samples were separated on 4-12% SDS-PAGE gradient gels (Invitrogen) and transferred onto nitrocellulose. The following antibodies and dilutions were used in this study for Western analysis: pan-lamin A/C (Cell signaling 2032; 1:1000), desmin (Abcam 15200; 1:25000), pERK1/2 (Cell Signaling 9101; 1:500), ERK1/2 (Santa Cruz sc-94; 1:2500), connexin 43 (Invitrogen 71-0700; 1:250), α-tubulin (Cell Signaling 2125; 1:1000), Cx43 NT1 and Cx43 CT1 (gifts from Paul Lampe, FHCRC, Seattle, WA; 1:1000). pERK/ERK ratio was quantified utilizing the "Measure" tool in ImageJ (NIH) to calculate pixel densitometry. CT1/NT1 ratio was quantified utilizing the “Rectangle Feature” in Odyssey software (LI-COR) to calculate pixel densitometry.

***Quantitative RT-PCR***

Hearts were homogenized using the TH homogenizer (Omni International) in RLT buffer (600 μL/30 mg tissue; Qiagen). Approximately 5 OD_260_ units of the RNA/protein mixture were used for the RNeasy kit with the remaining steps performed as per the manufacturer’s instructions (Qiagen). Reverse transcription reactions were performed with 2 µg total RNA using Superscript III (Invitrogen) at 42^o^C for 50 min. Quantitative RT-PCR (qPCR) samples were normalized against the housekeeping gene ribosomal protein, large, P0 (RPLP0; ARBP). qPCR primers are listed in the following or cited elsewhere: ANF Forward 5’-GAAAAGGCAGTCGATTCTGC-3’, Reverse: 5’-CAGAGTGGGAGAGGCAAGAC-3’; BNP ; RPLP0 Forward: 5’-TGTTTGACAACGGCAGCATTT-3’ Reverse: 5’-CCGAGGCAACAGTTGGGTA-3’.

***Echocardiography***

M-mode and Doppler imaging was performed to evaluate cardiac morphometry, systolic function, and myocardial performance index, which is calculated as the ratio of the sum of isovolemic contraction and relaxation time (IVCT + IVRT) to LV ejection time (LVET). An increase in MPI indicates that a larger fraction of systole is spent during isovolemic phases, which is the ineffective time fraction.

***ECG recordings***

Due to the small size of the *Lmna*^-/-^ mice, 5 min recordings were collected for anesthetized mice using 0.5% isoflurane with ECG leads placed at the modified lead II position of the chest cavity underneath the skin.

**Supplemental References:**

1. Sullivan T, Escalante-Alcalde D, Bhatt H, Anver M, Bhat N, et al. (1999) Loss of A-type lamin expression compromises nuclear envelope integrity leading to muscular dystrophy. J Cell Biol 147: 913-920.

2. Allegra S, Bouazza L, Benetollo C, Li JY, Langlois D (2005) A 7.1 kbp beta-myosin heavy chain promoter, efficient for green fluorescent protein expression, probably induces lethality when overexpressing a mutated transforming growth factor-beta type II receptor in transgenic mice. Transgenic Res 14: 69-80.
